# Characterizing Types of Human Mobility to Inform Differential and Targeted Malaria Elimination Strategies in Northeast Cambodia

**DOI:** 10.1038/srep16837

**Published:** 2015-11-23

**Authors:** Koen Peeters Grietens, Charlotte Gryseels, Susan Dierickx, Melanie Bannister-Tyrrell, Suzan Trienekens, Sambunny Uk, Pisen Phoeuk, Sokha Suon, Srun Set, René Gerrets, Sarah Hoibak, Joan Muela Ribera, Susanna Hausmann-Muela, Sochantha Tho, Lies Durnez, Vincent Sluydts, Umberto d’Alessandro, Marc Coosemans, Annette Erhart

**Affiliations:** 1 Department of Public Health, Institute of Tropical Medicine, Antwerp, Belgium; 2 School of International Health Development, Nagasaki University, Nagasaki, Japan; 3 Partners for Applied Social Sciences (PASS) International, Tessenderlo, Belgium; 4 Department of Biomedical Sciences, Institute of Tropical Medicine, Antwerp, Belgium; 5 National Center for Parasitology, Entomology and Malaria Control, Phnom Penh, Cambodia; 6 Amsterdam Institute for Social Science Research, Amsterdam, The Netherlands; 7 Global Fund to Fight AIDS, Tuberculosis and Malaria, Geneva, Switzerland; 8 Medical Research Council, Fajara, The Gambia; 9 London School of Tropical Medicine and Hygiene, London, UK

## Abstract

Human population movements currently challenge malaria elimination in low transmission foci in the Greater Mekong Subregion. Using a mixed-methods design, combining ethnography (n = 410 interviews), malariometric data (n = 4996) and population surveys (n = 824 indigenous populations; n = 704 Khmer migrants) malaria vulnerability among different types of mobile populations was researched in the remote province of Ratanakiri, Cambodia. Different structural types of human mobility were identified, showing differential risk and vulnerability. Among local indigenous populations, access to malaria testing and treatment through the VMW-system and LLIN coverage was high but control strategies failed to account for forest farmers’ prolonged stays at forest farms/fields (61% during rainy season), increasing their exposure (p = 0.002). The Khmer migrants, with low acquired immunity, active on plantations and mines, represented a fundamentally different group not reached by LLIN-distribution campaigns since they were largely unregistered (79%) and unaware of the local VMW-system (95%) due to poor social integration. Khmer migrants therefore require control strategies including active detection, registration and immediate access to malaria prevention and control tools from which they are currently excluded. In conclusion, different types of mobility require different malaria elimination strategies. Targeting mobility without an in-depth understanding of malaria risk in each group challenges further progress towards elimination.

Despite increasing understanding of the importance of ‘hot spots’ for malaria control and elimination^1^, comparatively less attention is being given to populations subgroups (‘hot pops’) that are vulnerable to malaria but cannot be easily located in a geographically confined area due to their mobility^2^. While human mobility has been addressed more extensively in relation to neglected tropical disease elimination^3^, immunization campaigns^4^ and HIV prevention^5^, it has, until recently, been largely neglected in malaria control and elimination^3^,^6^,^7^. Nevertheless, human mobility affects malaria transmission in several ways. Population movements expose individuals to a variety of health hazards to which sedentary populations are not exposed^8^ and it is often harder for those individuals to cope with the consequences of disease due, for instance, to language difficulties or a lack of familiarity with local health care. Moreover, the type of work highly mobile populations perform and the generally poorer working conditions can result in higher exposure to malaria vectors^6^,^9^,^10^, as has been reported on rubber plantations or in slash-and burn-agriculture^11^,^12^. Population movements may also result in non-immune individuals arriving in endemic areas or infected individuals seeking care in malaria-free regions, increasing difficulties in diagnostics and treatment^13^,^14^,^15^,^16^,^17^,^18^.

The importance of human mobility for malaria elimination was evident in previous elimination attempts where malaria re-emerged due to surveillance systems that failed to account for the movements of human populations^16^,^19^. Standard malaria control interventions implicitly operate on the assumption that individuals and subpopulations are registered and therefore easy to access. Current malaria control efforts typically target geographically stable groups: village malaria workers are assigned to highly endemic villages based on the administrative unit of the village/community; the distribution of long lasting insecticidal nets (LLIN) relies on intermittently updated census data and also indoor residual spraying (IRS) focuses on stable administrative villages; all of which consequently fail to account for mobile populations^10^,^17^,^20^,^21^. Moreover, in biomedical and clinical research, mobile individuals and mobile ethnic groups (such as e.g. nomadic Fula herders in SSA) are often purposefully excluded so as to minimize poor compliance to treatment or losses to follow-up. As a result, little is known about the effectiveness of standard interventions among these mobile populations. In the context of current malaria elimination targets, human mobility represents a major challenge for national control programs to further decrease malaria transmission^9^,^10^,^19^. Given the large sociocultural differences between various types of mobile populations, it can be expected that there is no standard way to address mobility in malaria control. In this sense, targeting pastoral nomads (i.e. pastoral herders in SSA) will require a radically different approach as compared to targeting national or international migrants, or commercial itinerant workers (i.e. street vendors and traders in border regions).

Several countries in the Greater Mekong Subregion are aiming to achieve malaria elimination within the next few decades^22^. However, residual transmission foci persist in forested areas such as the North-Eastern Cambodian province of Ratanakiri, that are largely populated by ethnic minority communities, often located at international borders and on the fringes of society, with increasing rural to rural migration to exploit new economic opportunities such as rubber plantations, gem mining and agriculture^23^. The aim of this study was therefore to characterize the different mobile groups in one such context and related those to vulnerability to malaria.

## Methods

### Study site and population

The study was conducted in the Cambodian province of Ratanakiri, traditionally populated by indigenous groups such as the Jarai, Kreung and Tompuon, also referred to as ‘ethnic minorities’, in contrast to the majority Khmer population that populates the rest of Cambodia. Indigenous subsistence strategies usually combine slash-and-burn agriculture with hunting, fishing, gathering forest products and small-scale trade. As distances between forest farms, rice fields and villages can be substantial (i.e. at several hours walking distance), most families maintain residences at each location and rotate from one place to another according to the agricultural cycle^24^. In addition to the indigenous populations, the rich resources of the province (forest, farmland, gems, etc.) have recently attracted many national and international investors mainly for large-scale rubber plantations. The latter require a large work force of mainly migrant workers from Cambodia’s rural and impoverished lowland provinces. In addition to plantation work, these rural-to-rural migrant Khmer farmers also seek irregular job opportunities on private farms or in construction and gem or gold mining in the area.

Malaria incidence in Ratanakiri is currently decreasing following intensified control measures, improved access to health care, socio-economic development and changes in the landscape, characterized by the replacement of the primary forests with rubber plantations, potentially impacting on vector populations^25^. Malaria transmission in Ratanakiri is perennial with two peaks, June-July and October-November, and the main vector is *Anopheles dirus*^25^,^26^.

In the public sector, malaria control measures (LLIN distribution, early diagnosis with RDT and treatment) are implemented through village malaria workers (VMWs) at community level. At the time of the study (2008–2012), the first line treatment was mefloquine-artesunate (A + M) for *Plasmodium falciparum* and chloroquine for *P. vivax*. In the private sector, Malarine™ branded blisterpacks of mefloquine-artesunate were sold alongside various drug cocktails (small plastic bags containing a variety of drugs such as antimalarials and antibiotics) and artemether injections^27^. Local indigenous groups traditionally consult diviners (traditional healers) to identify the cause of an illness, often in combination with various treatments from the public or private sectors^27^.

### Research strategy

**Figure 1:**
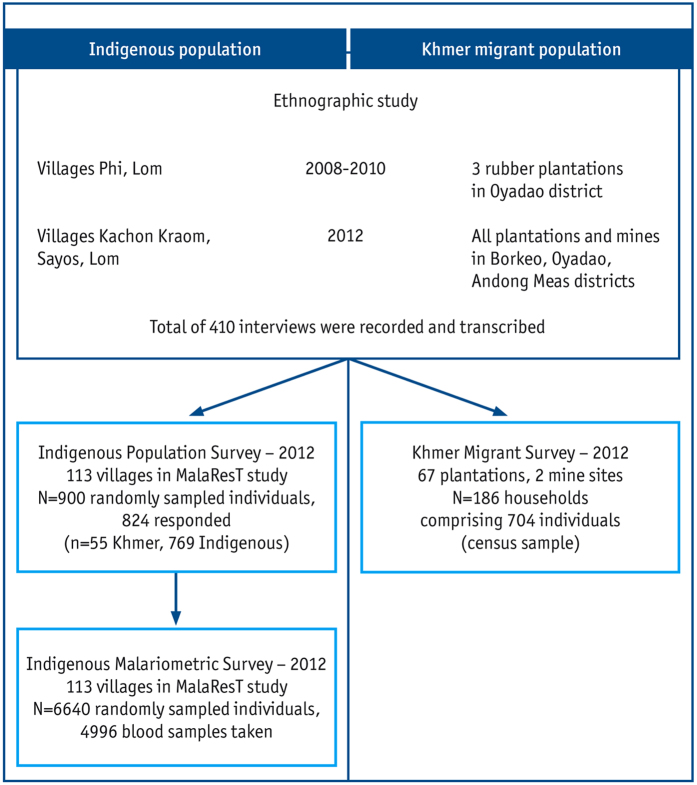
Flowchart of research strategy.

A parallel mixed-methods study design was chosen, using qualitative ethnographic research and quantitative survey research methods for complementarity (in standard annotation [qual + quan]^28^). Qualitative ethnographic data were collected in local communities and selected plantations to acquire an in-depth understanding of mobility patterns, living and working conditions, access to and use of malaria preventive measures and health care services of (i) Khmer migrant plantation workers and gem miners and (ii) local indigenous populations. Three additional surveys were aimed at quantifying the ethnographically assessed variables among these different populations. The ethnographic study and three surveys are described below and summarized in Table 1 and Fig. 1.

### Qualitative ethnographic study

The qualitative study was carried out in two phases: (i) A first exploratory qualitative phase was carried out between 2008 and 2010 in the villages of Phi and Lom (Oyado district), inhabited by the indigenous Jarai, and in three adjacent rubber plantations; (ii) In 2012, in-depth qualitative research was conducted in three villages included in a larger cluster randomized trial on the effectiveness of topical repellents as an added control measure to long-lasting insecticidal nets^25^ (NCT01663831, hereafter “MalaResT”). The Tompuon villages of Kachon Kraom (Voen Sai district) and Sayos (Lumphat district) and the Jarai village of Lom (Oyadao district) were selected. In addition, in Borkeo, Oyadao and Andong Meas districts, all identifiable plantations and gem mining sites were visited.

#### Data collection

Participant observation and in-depth interviewing were carried out. The former consisted of observations and reiterated informal conversations. Participant observation was primarily used as respondent-independent data collection tool to detect unforeseen variables and to contrast stated opinions with actual behavior.

#### Sampling

Multiple purposive sampling techniques were used. Following the principle of gradual selection, informants were theoretically selected (in accordance with emerging results/theory) and categorized in relation to relevant criteria (such as gender, age, locality, forest activities, previous experience with malaria, use of preventive measures, etc)^29^. In order to increase confidentiality with respondents and consequent reliability of the data, snowball sampling techniques (i.e. sampling using participants to identify additional cases) were used.

#### Data analysis

Qualitative data collection and analysis were performed concurrently and data analysis was an iterative process. Preliminary data were intermittently analyzed in the field, and preliminary results were then translated into the question guides for follow-up interviews. Analytic induction involved the iterative testing of theoretical ideas, which was used to refine and categorize themes grounded in the data^29^. This resulted in an analytical framework that was then systematically applied in the data analysis. Data were entered, managed, and analyzed in NVivo 9 Qualitative Data Analysis software (QSR International Pty Ltd. Cardigan UK).

### Khmer Migrant Survey

Three different risk groups –identified in the ethnographic study- were targeted: (i) rural to rural migrants working on rubber plantations; (ii) rural to rural migrants working in self-exploited (gold/gem) mines; and (iii) migrants having initially worked in plantations, but currently working in other informal jobs within the same general location (i.e. working on fields, etc). All work sites with migrant mobile populations in the districts of Oyadao, Andong Meas and Borkeo were identified and included in the study.

#### Data collection and sampling

In each study site, all Khmer working households (HH) that could be located on the plantation were invited to participate in the survey. After oral consent, first all participating household leaders were asked questions about their expected duration of residence, land ownership, administrative registration, income and use and access to malaria prevention tools. Secondly, the sleeping arrangements (use, type and condition of bed net or hammock net, sleeping surface) of all members of each household were observed and recorded.

#### Data analysis

Data was entered in Epi Info 7 (CDC, Atlanta, GA, USA) and analysed in SPSS (IBM SPSS Statistics 19). Frequency tables for the main descriptive variables were produced.

### Indigenous Population Survey

#### Data collection and sampling

A random sample of 900 individuals from different households were selected from the population census of the 113 villages included in the MalaResT study in 2012 and invited to participate in the survey. After oral consent, all participants were interviewed on the number of different settlements accessed by the individual’s household (village homes, homes on slash and burn farms, homes on rice fields), the time spent in each location by household members, and their use of malaria prevention measures.

#### Data analysis

Data was entered in Epi Info 7 (CDC, Atlanta, GA, USA) and analyzed in SPSS (IBM SPSS Statistics 19). Descriptive statistics were performed for the main outcome variables.

### Indigenous Malariometric Survey

#### Data collection and sampling

In addition to the *Indigenous Population Survey*, a malariometric survey was conducted during the MalaResT study in October 2012 to determine malaria prevalence and related risk factors. A total of 6,640 individuals across the 113 study villages were randomly selected from the census file and invited to participate in the survey. Participants were interviewed on use of preventive measures and overnight stays at the farm plot hut, clinically examined, and blood sampled for microscopy and molecular detection of malaria parasites^25^,^26^.

#### Data analysis

Data was entered in MS Access and analyzed in SPSS (IBM SPSS Statistics 19) and R (R version 3.1.1, The R Foundation for Statistical Computing). Frequency tables for the main outcome variables were produced. Two-level logistic regression with a random intercept fitted to adjust for clustering at village level was used to calculate the odds ratio for the association between spending nights in the forest in the past month and malaria infection, adjusted for age and sex.

### Ethical considerations

The study protocol was approved by the Institutional Review Board of the Institute of Tropical Medicine in Antwerp (ITM) and the Ministry of Health, Cambodia. The interviewers followed the Code of Ethics of the American Anthropological Association (AAA). All interviewees were informed before the start of the interview about project goals, the topic and type of questions, the intended use of results for scientific publications as well as their right to refuse being interviewed, interrupt the conversation at any time or withdraw any given information during or after the interview. Anonymity was guaranteed and confidentiality of interviewees assured by assigning a unique code number to each informant. The interviewers sought oral consent from all interviewees. Oral consent was preferred because the act of signing one’s name when providing certain information can generate mistrust. The quantitative and malariometric surveys obtained additional ethical clearance from ethical committee of the University Hospital of Antwerp and the National Ethics Committee for Health Research in Cambodia. For the surveys, the study objectives and methodology were first explained to each family in Khmer and individual written informed consent was given by each participant, or by parents/guardians of children below 18 years of age. All methods were carried out in accordance with the approved guidelines

## Results

### Study participants

#### Qualitative study

In total, 410 interviews, including formal and informal conversations, were recorded and transcribed.

#### Indigenous Population Survey

Among the 900 randomly sampled individuals, 824 (91.6%) were reached and interviewed, among which 55 individuals were Khmer (6.7%) that had migrated to a local village. All other respondents were indigenous people, including the ethnicities Jarai, Kreung, Tompuon, Kachok, Kavet, Lao, Lon and Prov.

#### Khmer Migrant Survey

Among the 67 plantations and 2 gem-mining sites included in the survey, 186 Khmer household leaders were interviewed. Of these 186 households, 704 household members’ sleeping arrangements were recorded.

#### Indigenous Malariometric Survey

Among the 6,640 individuals selected from the census file, 4,996 (75%) participants were reached.

### Types of mobility

During the first phase of the ethnographic study, the following types of mobile groups were identified (Table 1): first, Khmer rural-to-rural migrants, including (*i*) *Permanent national rural to rural migration,* which describes Khmer farmers leaving their rural communities in lowland provinces of Cambodia to settle permanently in highland Ratanakiri and work on large-scale plantations or smaller farms; and (*ii*) *Seasonal/temporal national rural to rural migration*, referring to Khmer farmers temporarily working on plantations in Ratanakiri and then returning to their villages at the end of their contract. Secondly, indigenous population movements were identified that included mobility due to (*iii*) *indigenous multiple residence systems.* This translates into local indigenous population movements driven by subsistence requirements (e.g. sleeping at farms in the forest) and economic activities (e.g. spending nights in the forest for hunting). Indigenous population movement also included (*iv*) *cross-border mobility*, meaning movement of indigenous people across the Cambodian/Vietnamese border for economic (buying or selling products across the border) and/or social (visiting relatives) reasons.

### Vulnerability of Khmer rural to rural migrants to malaria infection

#### Mobility

More than half (56.5%) of the Khmer households working on plantations, all households working in gem-mining sites and most households (70.6%) headed by independently working Khmer migrants were categorized as “permanent rural to rural migration”, reportedly having come to Ratanakiri to work for an indefinite period of time (Table 2). Among the Khmer plantation workers, 43.5% could be categorized as seasonal rural to rural migrants, with a median working time of 5 months (IQR 16, range 1 to 108 months); a figure that went up to 48 months among miners (IQR 54, range 1 to 240 months). Traditionally, the Khmer New Year (April) marked the time point at which the Khmer migrant plantation workers returned home and new migrants came to replace them.

#### Socio-economic vulnerability

Overall, only 7.0% of all Khmer migrant households owned some land for small subsistence farming in Ratanakiri, while the majority did not own land at all (53.2%), especially among the miners (83.8%). Miners also seldom owned land in their home province (8.1%) compared to plantation or other workers (respectively, 52.2% and 38.2%; Table 2). On plantations, the majority of Khmer workers (79.1%) were not officially registered in Ratanakiri; though most of them (84.6%) reported being registered in their home villages. Local official registration was higher among Khmer migrants working independently on private farms (55.9%) and on mining sites (67.6%). The Khmer migrant households’ monthly income ranged between 0 and 800.000 riel (200 USD) with a median of 250.000 riel (62.5 USD) (Table 2). and half of households reported not being able to save any of this money.

#### Access to health care and malaria prevention

Only 5.4% of Khmer migrant respondents knew about the existence of VMW’s in the nearby indigenous villages, (Table 2); and only 2.7% knew the actual VMW of the village where their plantation/mine was located. The lack of administrative registration among the majority of Khmer migrants resulted in the majority of households never having received a bed net from the NMCP in Ratanakiri (66.7%), especially those households working on plantations (91.3%). Moreover, a majority (76.3%) of Khmer migrants reported never having received a bed net from the NMCP in their home province. Nevertheless, the majority of the households did bring bed nets (both treated and non-treated) to their new work locations (89.2%), and most of them considered the number of bed nets they brought themselves sufficient to protect all household members (71.7%) (Table 2).

When considering individual malaria prevention among household members of the migrant households, almost all (91.1%) respondents reported to use a bed net, usually while sleeping on mats, or on rare occasions, in hammocks (Table 3). Bednets were usually non-treated and bought from the market (74.7%) and over half of the respondents were sleeping under nets that were observed to be torn (i.e. defined as nets with rips and tears).

### Indigenous population in-country mobility

#### Mobility

Mobility among the indigenous population is linked to the tradition of slash-and-burn agriculture. According to the ethnographic study, indigenous families combine sleeping in village homes (traditionally longhouses) with one or several homes at their farms or rice fields in the forest. According to the Indigenous Population Survey (Table 4), most respondents (93.2%) had one or more forest farms, as well as a house on the farm(s) (82.5%). The rainy season, especially during the harvest months, is the most work intensive season for indigenous farmers, often requiring them to sleep in plot huts at their farms in the forest. Indeed, 61.2% reported sleeping at their farms during the malaria season (rainy season which extends into harvest months). In addition to forest farms, 44.1% of respondents reported having a wet rice field, and of those, the majority (67.5%) had built a house on that rice field where they would also be sleeping during the malaria season (53.2%).

The village home is mostly used during the dry season when the most labor-intensive work at the field is over and when farmers rest and have their annual ceremonies and celebrations in the village home. Accordingly, only 33.1% of all respondents reported always sleeping in the village during the rainy season, as opposed to the 72.5% that always sleep in the village during the dry season.

Forest farms are usually reached by foot, and can be located as far as 10 kilometers from the village, making it difficult to transport the necessary materials, such as food, water, mats, and bed nets, back and forth between village and farm (Table 4). The majority (74.8%) of respondents reported moving between farms and fields without stopping over in the village, making it even more difficult to carry around sufficient bed nets or hammock nets to protect all family members who go on the journey. However, due to the recent national malaria control policy (since 2011) of distributing one bed net per person, the largely officially registered indigenous families seem to have sufficient bed nets from the NMCP in combination with non-impregnated nets bought from the market to spread them out over several locations, as multiple family members sleep under one net at different sleeping places. Over half of respondents therefore report to permanently keep some bed nets at their farms (58.1%) and rice fields (50.6%), and the majority reports to usually have a bed net to use at farms (73.3%) and rice fields (77.6%) (Table 4).

#### Deep forest activities

67.2% of the indigenous population engaged in deep forest activities. These consist of hunting, gathering forest products such as bamboo or wood and other activities in the uncleared forest where people potentially sleep in an improvised way (e.g. in hammocks). 23.6% reported spending nights in the deep forest (Table 4). The malariometric survey indicated a significant relationship between spending nights at the plot hut on the forest farm in the last month and increased odds of malaria infection (OR 1.66, 95% CI 1.20-2.28, p = 0.002). There was a trend towards increased odds of malaria associated with spending nights in the deep forest in the last month, though there was only weak statistical evidence for this association (OR 1.35, 95% CI 0.94-1.98, p = 0.10) (Table 5).

#### Access to health care and malaria prevention

Unlike the Khmer migrants, most local indigenous villagers knew the local VMW since he/she was chosen from the indigenous community; only 22.8% of indigenous respondents had never visited the VMW (Table 4). Of those who had visited the VMW for a RDT, 71.5% reported the test to have been positive for malaria, and most of those positive cases (81.6%) received treatment from the VMW.

### Indigenous population cross-border mobility

#### Mobility

According to the ethnographic study, in the three Jarai villages at the border area, the indigenous territory consists of communities spread over both sides of the border. Short-term cross-border human population movements occurred frequently as the Vietnamese and Cambodian Jarai living within the border communes are allowed to cross the border without permits for one-day stays. It was primarily Cambodian Jarai that crossed the border into Vietnam to pursue economic activities, namely to sell agricultural products and/or to buy products at local Vietnamese stores and markets to take back to Cambodia for resale in small privately owned shops. Longer stays, for which community-level permits and justifications are needed, were generally driven by wanting to seek care in Vietnamese health centers (also for malaria treatment), to visit relatives, and to attend ceremonies. Given the greater economic growth in Vietnam, few Vietnamese Jarai venture into Cambodia for business, but do cross the border for celebrations and other family obligations, as well as to cultivate land in the forest for slash and burn agriculture unavailable in Vietnam.

#### Malaria prevention

When visiting relatives or other relations across the border, it is not usual to take bed nets when spending nights at hosts’ houses across the border. The host family often do not have enough nets to share and rules of hospitality do not require the host to provide bed nets to visitors. However, families that visit once or twice a year and only for ceremonies do so mostly during the dry season.

## Discussion

Our study characterized the different structural types of human mobility in Ratanakiri, and their differential risk and vulnerability towards malaria exposure, clearly showing the need for different and adapted malaria prevention and control measures among the groups that are, nevertheless, usually jointly categorized under “mobile populations”. Among the local indigenous population, in contrast to the Khmer migrants, LLIN coverage was very high. However, this LLIN distribution was based solely on the administrative village setting, thereby failing to take into account local forest farmers’ multiple residence system which entails prolonged stays in the forest farms/fields and increases their exposure to the known early outdoor biting behavior of sylvatic *An. dirus*^12^,^21^. Control measures targeting the forest farms, such as additional LLINs and/or other innovative tools, such as spatial repellents or toxic sugar bait traps, can be expected to have a further impact on malaria^30^,^31^. Types of human population movements, taking place on a relatively local level, easily go undetected, as illustrated by the multiple residence system among the indigenous Jarai. They could, however, be key to malaria elimination due to the mentioned exposure and in relation to difficulties with uptake of elimination strategies such as MDA, where these populations may easily be missed. More irregular and individual mobility related to the family visits, trade, the exploitation of forest products, including logging, require different tools and strategies, examples of which could be long-lasting insecticidal hammocks^32^,^33^,^34^,^35^,^36^, topical repellents or insecticide treated clothing^37^,^38^.

With regard to cross-border mobility, while the current borders of the nation-states divide traditional indigenous territory, their social and economic networks require cross-border movements. These cross-border movements can increase local populations’ vulnerability to malaria as no bed nets are transported during these visits and hosts generally do not offer them to their guests. In addition, the differential application, in method and intensity, of malaria control activities on either side of nations’ borders has been shown to potentially lead to the maintenance of the parasite reservoir by a constant supply of new strains^39^.

Additional difficulties for malaria control among indigenous populations include language barriers, historically complicated inter-ethnic relations, the power imbalance observed between local communities and large companies that are currently converting traditional territories into plantations. All of these factors can be hypothesized to compel the most vulnerable groups among indigenous populations, in terms of socio-economic status and social integration, to retreat deeper into remote forested areas, further increasing their exposure/risk and decreasing their accessibility to health services.

The Khmer rural-to-rural migrant group, mostly active on rubber plantations and in mines, represents a fundamentally different social group that increasingly develops as a parallel “social territory” to that of the indigenous populations. The continuous influx of these lowland Khmer constitutes a new potential risk group for malaria. These migrants are often moving from lowland non-endemic provinces, and are therefore more vulnerable to malaria infection due to their limited awareness of malaria prevention and treatment as well as low acquired immunity, in contrast to the indigenous populations, that have good access to malaria diagnostic and treatment through the VMW system and have a higher level of immunity. Khmer migrants were seldom reached by LLIN-distribution campaigns since they remained largely unregistered, and were also unaware of the local VMW-system due to poor social integration locally. Moreover, most plantation workers and miners purchased non-treated nets, the majority of which were already damaged at the time of study, providing less protection against mosquito bites.

The temporal migration from high to low-endemic areas, as large numbers of Khmer migrants return to their home provinces after their work has ended, can result in imported infections^16^ or re-introduce malaria where transmission had ceased, which is especially the case in areas of high receptivity (i.e. the historical potential for vector transmission that determines the severity of local onward transmission)^16^. Khmer temporal migrants therefore require different malaria control strategies than local indigenous populations, such as the active detection and registration of those workers and immediate access to malaria prevention and control tools from which they are currently excluded.

The strength of this study lies in its mixed-methods design that allowed the integration of qualitative and quantitative data, producing consistent and complementary findings across different datasets. The triangulation design reduced the risk of bias as it allows the validation of different findings observed across different methods. An additional strength was the interdisciplinarity, which enabled linking different types of local mobility patterns to malaria risk as measured by malariometric data. The ethnographic data collection provided richer contextual data than would have been possible to obtain through standard questionnaires during malariometric surveys. Furthermore, the ethnographic component explicitly informed the development of the malariometric survey by a prior identification of all possible local risk factors/behaviors. The main limitation of the study was the lack of malariometric data on migrant workers in rubber plantations and gem mines that could have assessed the relevance of their exposure in relation to malaria risk. This should be further assessed in population-based studies including the different groups at risk. In addition, due to the rapid economic changes in the region and the continuous exploitation of new plantations, some plantations may have been missed. We do not expect, however, that including additional plantations would have changed the presented results.

## Conclusion

**Figure 2:**
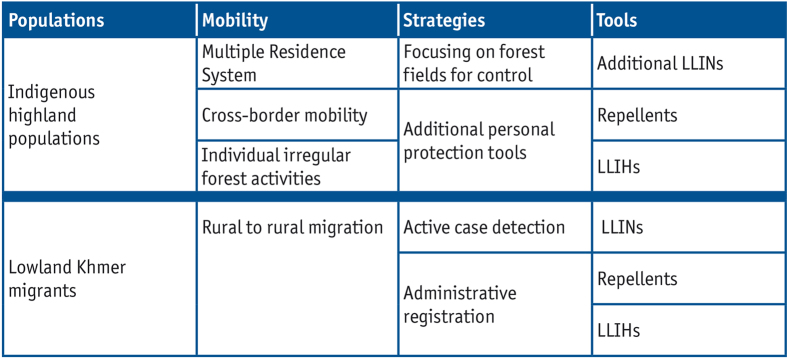
Malaria vulnerability by types of mobility.

Different types of mobility require different malaria control and, ultimately, elimination strategies (Fig. 2). In addition, targeting mobility without an in-depth understanding of malaria risk in each group can lead to wasted efforts and resources. Further studies should be systematically embedded into national malaria control and elimination programs in order to identify the different type(s) of mobile populations at stake and enable the development of a theoretical framework for optimizing the delivery of effective malaria control measures to those most in need.

## Additional Information

**How to cite this article**: Peeters Grietens, K. *et al.* Characterizing Types of Human Mobility to Inform Differential and Targeted Malaria Elimination Strategies in Northeast Cambodia. *Sci. Rep.* **5**, 16837; doi: 10.1038/srep16837 (2015).

## Figures and Tables

**Table 1:** Methods used.

Qualitative methods		
*Population*		
*Ethnographic Study*	Indigenous populations and Khmer migrant workers	
**Quantitative methods**	*Population*	*N*
*Khmer Migrant Survey*	Khmer migrants in rubber plantation, gem/gold miners, or other informal activities	n = 186
*Indigenous Malariometric Survey*	Indigenous populations	n = 6,640
*Indigenous Population Survey*	Indigenous populations	n = 824

**Table 2:** Khmer Migrant Survey–Household leaders (N = 186).

	Plantation (N = 115)	Mines (N = 37)	Other (N = 34)	Total (N = 186)
	n (%)	n (%)	n (%)	n (%)
Duration of stay				
** **Indefinite	65 (56.5)	37 (100)	24 (70.6)	126 (67.7)
** **Definite	50 (43.5)	0 (0)	10 (29.4)	60 (32.3)
** **Median stay (months)	5	48	24	12
Land ownership				
** **HH° owns land in Ratanakiri	5 (4.3)	3 (8.1)	5 (14.7)	13 (7.0)
** **HH owns land in home province	60 (52.2)	3 (8.1)	13 (38.2)	76 (40.9)
** **HH does not own land at all	51 (44.3)	31 (83.8)	17 (50.0)	99 (53.2)
Administrative registration				
** **Registered locally	21 (18.3)	25 (67.6)	19 (55.9)	65 (34.9)
** **Not registered locally	91 (79.1)	11 (29.7)	15 (44.1)	117 (62.9)
** **Don’t know	3 (2.6)	1 (2.7)	0	4 (2.2)
** ***Amongst those not registered:*	*N* = *91*	*N* = *11*	*N* = *15*	*N* = *117*
Registered in home village	77 (84.6)	6 (54.5)	13 (86.7)	77 (81.9)
Income				
** **Median monthly income (riel)	300.000R	200.000R	300.000R	250.000R
** **Not able to save any money	47 (40.9)	28 (75.7)	17 (50.0)	92 (49.5)
** **Manager deducts cost of food from salary	35 (30.4)	1 (2.7)	2 (5.9)	38 (20.4)
VMW				
** **Knows local VMW	3 (2.6)	1 (2.7)	1 (2.9)	5 (2.7)
** **Knows of existence VMW	7 (6.1)	1 (2.7)	2 (5.9)	10 (5.4)
** **Did not know of existence	108 (93.9)	36 (97.3)	32 (94.1)	176 (94.6)
Bed nets				
** **Never received program net in home province	76 (66.1)	28 (75.7)	20 (58.8)	142 (76.3)
** **Never received program net in visiting province	105 (91.3)	18 (48.6)	19 (55.9)	124 (66.7)
** **Owned bed nets upon arrival	99 (86.1)	36 (97.3)	31 (91.2)	186 (89.2)
** ***Amongst those who owned nets upon arrival*	*N* = *99*	*N* = *36*	*N* = *31*	*N* = *186*
** **Considered bed nets owned upon arrival to be sufficient	67 (67.7)	29 (80.6)	23 (72.2)	119 (71.7)

°HH = household leaders.

*Includes Khmer workers who initially worked on plantations but now work other informal jobs in the same area, such as labourers on private farms.

**Table 3:** Khmer Migrant Survey: All Household Members (N = 704).

	Plantation *N* = *374*	Mines *N* = *159*	Other *N* = *171*	Total *N* = *704*
	n (%)	n (%)	n (%)	n (%)
**Sleeping habits**				
Sleeps on mat	356 (95.2)	159 (100)	163 (95.3)	678 (96.3)
Sleeps in hammock	15 (4.0)	0	6 (3.5)	21 (3.0)
Missing	3 (0.8)	0	2 (1.2)	5 (0.7)
Uses a bed net	325 (86.9)	157 (98.7)	159 (93.0)	641 (91.1)
*Amongst bed net users:*	*N* = *325*	*N* = *157*	*N* = *159*	*N* = *641*
Net type				
** **NTN	269 (82.8)	92 (58.6)	118 (74.2)	479 (74.7)
** **ITN/LLIN	55 (16.9)	65 (41.4)	40 (25.2)	160 (25.0)
** **Missing	1 (0.3)	0	1 (0.6)	2 (0.3)
Net status				
** **Intact net	88 (27.2)	95 (60.5)	104 (65.4)	287 (44.8)
** **Torn net	237 (72.9)	62 (39.5)	54 (34.0)	353 (55.1)
** **Missing	0	0	1 (0.6)	1 (0.2)

**Table 4:** Indigenous Population Survey (N = 824).

	n (%)
**Forest farms**	
Has forest farm(s)	768 (93.2)
*Amongst those who have farm*(*s*)	*N* = *768*
Has a house on farm(s)	633 (82.5)
Sleeps at farm during malaria season	470 (61.2)
*Amongst those who have a house on farm*(*s*)	*N* = *633*
Has a bed net to use at farm	464 (73.3)
Brings back net back and forth from village	96 (15.2)
Keeps bed nets at farm	368 (58.1)
Transportation to farm (*multiple responses possible*)	
On foot	677 (88.2)
By motorbike	346 (45.1)
By boat	44 (5.7)
By bicycle	25 (3.3)
Other	7 (0.9)
**Wet Rice Fields**	
Has wet rice field(s)	363 (44.1)
*Amongst those who have wet rice field*(*s*)	*N* = *363*
Has a house on field(s)	245 (67.5)
Sleeps at field during malaria season	193 (53.2)
*Amongst those who have a house on rice field*(*s*)	*N* = *245*
Has a bed net to use at field	190 (77.6)
Brings back net back and forth from village	66 (27.0)
Keeps bed nets at field	124 (50.6)
Transportation to field (*multiple responses possible*)	
On foot	323 (89.0)
By motorbike	121 (33.3)
By boat	23 (6.3)
By bicycle	4 (1.1)
Other	6 (1.7)
**Village**	
Has a village house	755 (91.6)
*Amongst those who have village house*	*N* = *755*
Always sleeps in village during dry season	597 (72.5)
Always sleeps in village during rainy season	273 (33.1)
**Mobility between farms–fields–village**	
Moves between farms and/or fields without stopping in the village	529 (74.8)
*Amongst those who move between farms and/or fields*	*N* = *529*
Moves often between farm(s)-field(s) (1–7 times/ week)	281 (53.1)
Moves sometimes between farm(s)-field(s) (1–15 time/month)	115 (21.7)
Never moves between farm(s)-field(s)	116 (21.9)
Missing	17 (3.2)
*Items carried between farm-field-village* (*multiple responses possible*):	
Bed nets	69 (8.4)
Hammock nets	38 (4.6)
Sleeping mats	62 (7.5)
Blanket	74 (9.0)
Food	466 (56.6)
Water	116 (14.1)
Cooking material	185 (22.5)
Hunting / fishing material	21 (2.5)
Logging material	259 (31.4)
Farming material	356 (43.2)
**Forest activities**	
Goes to deep forest	554 (67.2)
*Amongst those who go to deep forest*	*N* = *554*
Stays overnight at forest	131 (23.6)
Goes hunting or fishing in forest	288 (52.0)
Gathers forest products (fruits, vegetables, firewood, honey, etc)	477 (86.1)
Goes logging in forest	151 (27.3)
**VMW accessibility and treatment**	
Never visited the VMW before	188 (22.8)
Have visited the VMW before	631 (76.6)
Missing	5 (0.6)
*Amongst those who have ever visited the VMW before*	*N* = *631*
VMW not available during last visit	28 (3.4)
RDT was not available during last visit to VMW	108 (13.1)
RDT was available during last visit to VMW	495 (60.1)
*Amongst those who had an RDT during last visit to VMW*	*N* = *495*
Negative test during last visit	141 (28.5)
Positive test during last visit	354 (71.5)
*Amongst those who had a positive RDT during last visit to VMW*	*N* = *354*
Received treatment from VMW	289 (81.6)
Received treatment from public sector (HC, hospital)	45 (12.7)
Received treatment from private sector	13 (3.7)
Received treatment from other	7 (2.0)

**Table 5:** Association between plot hut (farm or field) and forest overnight stays in the last month and malaria infection (all species).

	Total	Total malaria infected (PCR)	Malaria prevalence %	OR*	95% C.I.	p-value
**Plot hut overnight stay in last month**						
Yes	2727	166	6.0	1.66	[1.21; 2.28]	0.002
No	2238	77	3.0	Ref.	−	
**Forest overnight stay in last month**						
Yes	800	59	7.0	1.36	[0.94; 1.98]	0.10
No	4165	184	4.0	Ref.	−	

^*^Adjusted for age and sex, and clustering by village.
